# Parsing the contributions of negative affect vs. aversive motivation to cognitive control: an experimental investigation

**DOI:** 10.3389/fnbeh.2023.1209824

**Published:** 2023-09-18

**Authors:** Qian Yang, ShuangQing Si, Gilles Pourtois

**Affiliations:** ^1^Institute of Brain and Psychological Sciences, Sichuan Normal University, Chengdu, China; ^2^Cognitive and Affective Psychophysiology Laboratory, Department of Experimental Clinical and Health Psychology, Ghent University, Ghent, Belgium

**Keywords:** conflict processing, aversive motivation, negative affect, feedback contingency, punishment

## Abstract

**Introduction:**

Punishment is a powerful drive that fosters aversive motivation and increases negative affect. Previous studies have reported that this drive has the propensity to improve cognitive control, as shown by improved conflict processing when it is used. However, whether aversive motivation *per se* or negative affect eventually drives this change remains unclear because in previous work, the specific contribution of these two components could not be isolated.

**Methods:**

To address this question, we conducted two experiments where we administered the confound minimized Stroop task to a large group of participants each time (*N* = 50 and *N* = 47 for Experiment 1 and 2, respectively) and manipulated punishment and feedback contingency using a factorial design. These two experiments were similar except that in the second one, we also measured awareness of feedback contingency at the subjective level. We reasoned that cognitive control would improve the most when punishment would be used, and the contingency between this motivational drive and performance would be reinforced, selectively.

**Results:**

Both experiments consistently showed that negative affect increased at the subjective level when punishment was used and the feedback was contingent on task performance, with these two effects being additive. In Experiment 1, we found that when the feedback was contingent on task performance and punishment was activated, conflict processing did not improve. In Experiment 2, we found that conflict processing improved when punishment was contingent on task performance, and participants were aware of this contingency.

**Discussion:**

These results suggest that aversive motivation can improve conflict processing when participants are aware of the link created between punishment and performance.

## Introduction

Punishment can improve and sharpen cognitive control during conflict processing (Stürmer et al., 2011; Yang and Pourtois, 2018; Yang et al., 2019), probably because participants are motivated to avoid the unpleasant experience associated with this incentive. In agreement with this assumption, in several previous studies (see Yang and Pourtois, 2018; Yang et al., 2019, 2022), we used a (small) monetary loss as negative feedback contingent on task performance and found that participants showed improved cognitive control (at the level of conflict processing or conflict adaptation) as a result of it. It corresponded to a gain in conflict processing, mostly expressed in RTs when punishment feedback was used, and this facilitation was observed at the level of the congruency effect or conflict adaptation effect. Moreover, this manipulation usually led to increased levels of negative affect at the subjective level. However, the cause or origin of this gain in cognitive control remains unclear because punishment (or its prospect) activates aversive motivation (Lindström et al., 2013; Yang et al., 2019, 2022; Liao et al., 2020; Yee et al., 2022) while it also increases negative affect concurrently (Erdle and Rushton, 2010) and these two components are not fully overlapping with each other. As a matter of fact, there are close ties between aversive motivation and negative affect (Cacioppo and Berntson, 1994). To some extent, while punishment can be seen as a powerful drive that activates aversive motivation, negative affect can be considered as the logical and adaptive consequence of this prior activation (Cooper, 2019), especially if this punishment can in fact be hardly avoided (e.g., when a stringent response deadline is used, as adopted in our previous studies).

According to the affective-signaling hypothesis (Dignath et al., 2020), negative affect, but not aversive motivation *per se*, is the key ingredient that drives cognitive control in general, and conflict processing more specifically. In line with this idea, there have been several studies showing that conflict adaptation is increased with the induction of negative mood (van Steenbergen et al., 2010; Schuch and Koch, 2015; Schuch et al., 2017; Schuch and Pütz, 2018), for which aversive motivation is not directly involved presumably. However, other studies have reported that conflict adaptation is increased when punishment (i.e., monetary loss) contingent on performance is used (see Yang and Pourtois, 2018; Yang et al., 2019). In this situation, aversive motivation is elicited and as explained here above, negative affect is likely the consequence rather than the cause of this change in cognitive control. For example, we previously manipulated aversive motivation at the block level. Participants received either a positive or negative feedback that was contingent on task performance during the Stroop task (Yang and Pourtois, 2018). Importantly, this negative feedback was converted to a small monetary loss in these blocks (negative blocks), while in others (neutral blocks), it was not. Results showed that in negative blocks, participants reported increased negative affect (as well as decreased positive affect) at the subjective level compared to neutral blocks. Importantly, cognitive control (i.e., conflict adaptation) improved in negative compared to neutral blocks, suggesting that it benefited from aversive motivation (Yang and Pourtois, 2018). Moreover, a recent study showed that negative affect resulting from the processing of (unrelated) emotional words, which is not connected to aversive motivation, is not sufficient to enhance cognitive control (Bognar et al., 2023). Hence, aversive motivation may have a stronger impact on cognitive control (e.g., conflict processing) than negative affect (Inzlicht et al., 2015). Importantly, we could show that this impact truly reflected a gain in conflict processing because both the N2 event-related potential (ERP) component and mid-frontal theta (MFT) power (Cavanagh and Frank, 2014) increased in negative compared to neutral blocks (Yang et al., 2019).

To further corroborate the (preferential) link between conflict processing and aversive motivation, we recently performed a study where this punishment feedback was selectively paired with either congruent or incongruent trials, using again a block design (Yang et al., 2022). Results showed that the congruency effect decreased when punishment was paired with incongruent trials while conflict adaptation improved instead when punishment was paired with congruent trials. Tentatively, this dissociation suggests that feedback contingency might have different effects on the proactive (expressed by the congruency effect) and reactive components (expressed by the conflict adaptation effect) of cognitive control. Moreover, this pattern of results led us to believe that aversive motivation, rather than negative affect *per se*, could be the key ingredient necessary for improving and sharpening cognitive control, in a context sensitive manner. It is noteworthy that in our study (Yang et al., 2022), “by design,” punishment could not be avoided (see also Yang and Pourtois, 2018). Even when participants objectively speeded up response times (for correct response), the use of a stringent and adaptive (updated on a trial-by-trial basis) cutoff to discriminate fast (leading to positive feedback) from slow responses (leading to negative/punishment feedback) made it impossible to decrease punishment probability. However, the participants were not aware of this RT cutoff and its dynamic change or update over time. Hence, in this peculiar situation, we found that conflict processing improved the most when punishment was paired with incongruent trials while conflict adaptation basically improved when conflict resolution was not jeopardized by it (i.e., when it was paired with congruent trials, see Yang et al., 2022; see also Yee et al., 2022).

Accordingly, these previous results point to aversive motivation, rather than negative affect, as the main component necessary to improve cognitive control temporarily. However, to further substantiate this claim, it remains to be shown that when punishment is not contingent on task performance, negative affect is generated in turn, yet it does not necessarily lead to a gain in cognitive control. Surprisingly, few studies have tested this important assumption. In practically all studies published so far, punishment was contingent on task performance (Stürmer et al., 2011; Yang and Pourtois, 2018; Choi and Cho, 2020; Yang et al., 2022, Experiment 2). To the best of our knowledge, only Stürmer et al., 2011 (Experiment 1) used monetary incentives that were randomly presented in between trials and found that conflict adaptation was unaffected by them. Hence, contingency could be an important boundary condition for the improvement of cognitive control because it spurs aversive motivation. Indeed, when punishment is contingent on (task) performance, aversive motivation is activated in turn, which can improve and guide information processing, thereby resulting in a gain in conflict processing (Yee et al., 2022).

The goal of our study was to test this important assumption. To this end, we used the confound minimized Stroop task to measure conflict processing (Braem et al., 2019). Crucially, negative affect (resulting from the encounter of punishment) and aversive motivation (resulting from punishment contingent on performance) were manipulated independently from each other using a factorial design. We hypothesized that the congruency effect, which is a standard index of cognitive control in the literature (Egner, 2008), should improve the most when aversive motivation would be activated, and the (punishment) feedback would be made contingent on task performance. Alternatively, if negative affect, but not aversive motivation, is what drives cognitive control during conflict processing, then it should improve the most when punishment is involved, yet irrespective of feedback contingency. To adjudicate between these two hypotheses, we conducted two experiments that used a similar factorial design enabling us to disentangle effects of negative affect from aversive motivation. The key difference between them is that we additionally measured at the subjective level awareness of feedback contingency in Experiment 2 because meta-control might be an important factor to explain the putative modulation of cognitive control by aversive motivation (Yee et al., 2022).

## Experiment 1

### Methods

#### Participants

Based on our previous work (Yang and Pourtois, 2018), we ran a power analysis (using G*power) that indicated that 50 participants had to be included in the sample, when an effect size of *d* = 0.45 with a power of 80% were set, and a 2 × 2 × 2 within-subject design was used. Fifty-three Dutch-speaking participants took part in this experiment. Three of them were excluded because of low accuracy (i.e., smaller than 60%). As a result, fifty participants were included in the final data analyses (10 males, mean age = 21.44 years, standard deviation (SD) = 4.18). They all had normal or corrected-to normal vision, and no history of psychiatric or neurological disorders. On average, participants lost 2 Euro during blocks with punishment feedback and were compensated 12 Euro for their participation.

#### Stimuli and task

We used a color-word Stroop task to measure conflict processing. The Stroop stimuli consisted of four words (in Dutch) (“rood”/red, “blauw”/blue, “groen”/green, or “geel”/yellow; font size, 30 points) presented in one out of four possible colors (red, RGB: 255, 0, 0; blue, RGB: 0, 176, 240; green, RGB: 0, 255, 0; yellow, RGB: 255, 255, 0). To rule out contingency learning, a four-alternative forced choice (4-AFC) task was used (Schmidt and Weissman, 2014), where two pairs of S-R were created arbitrarily to balance congruent and incongruent trials. Each pair consisted of two words and two colors such that incongruent trials were created for the (incompatible) word-color association within each pair, but not across pairs however. According to this rule, 8 stimulus types were created in total (instead of 16 if all combinations were constructed), corresponding to 4 stimuli for congruent trials and 4 stimuli for incongruent trials. Each word was presented equally often in the congruent and incongruent color in each block with each mapping (Mordkoff, 2012). To rule out feature repetitions across successive trials, the stimuli were systematically alternated across them to ensure that there was not stimulus (or response) repetition for both goal-relevant (color) and goal-irrelevant (meaning) dimension. Each trial started with a fixation cross that was used as inter-trial interval (ITI), with a mean duration of 500 ms (range: 400–600 ms). After this, the Stroop stimulus was presented in the middle of the screen for 1000 ms or until a response was given, followed by a blank screen shown for 700 ms. Finally, either a negative feedback (black cross) or a positive feedback (black tick mark) was provided. On each trial, participants were instructed to identify the color of the word (i.e., color naming task) as fast and accurate as possible by using four predefined keys of a response box. These four keys corresponded to four colors (i.e., red, blue, green, yellow). More specifically, they used their left middle finger to respond to red color, left index finger to blue color, right index finger to green color, and right middle finger to yellow color.

#### Procedure

**FIGURE 1:**
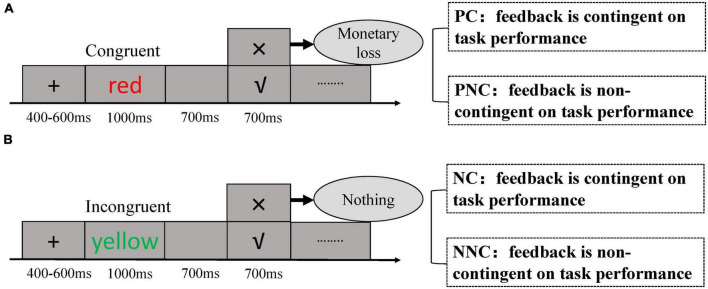
Experimental procedure. Each trial started with a fixation cross (that lasted on average 500 ms), followed by a Stroop stimulus. A blank screen was then presented, before either a negative or positive feedback appeared. **(A)** Punishment conditions. Punishment-contingency session (PC): the feedback was contingent on performance, and each negative feedback was converted to monetary loss. Punishment-non contingency session (PNC): the feedback was not contingent on performance and each negative feedback was converted to monetary loss. **(B)** Neutral conditions. Neutral-contingency session (NC): the feedback was contingent on performance, and each negative feedback was not converted to monetary loss. Neutral-non contingency session (NNC): the feedback was not contingent on performance, and each negative feedback was not converted to monetary loss.

We manipulated both Feedback type (punishment vs. neutral) and Feedback contingency (contingent vs. non-contingent) concurrently. Consequently, the experiment consisted of four conditions: Punishment-contingency (PC, see Figure 1A-PC), Punishment-non contingency (PNC, see Figure 1A-PNC), Neutral-contingency (NC, see Figure 1B-NC), and Neutral-non contingency (NNC, see Figure 1B-NNC). More specifically, for the PC and NC conditions, the feedback (either negative or positive) was based on actual performance. A negative feedback (black cross) was provided if the response was incorrect or too slow (i.e., slower than an arbitrary time limit; see below)^1^. A positive feedback (black tick mark) was provided if the response was correct and fast enough (i.e., below this time limit; see Figure 1). In comparison, for the PNC and NNC conditions, the feedback was random and independent of actual performance. Of note, the proportion of negative and positive feedback was comparable to the PC and NC conditions (see Table 1). When punishment was involved (PC and PNC), negative feedback was converted into monetary loss (2 cents), whereas when it was not (NC and NNC), there was no consequence. Regarding the adaptive RT cutoff used to separate fast from slow responses (and positive from negative feedback), it was based on our own previous studies (see Yang and Pourtois, 2018; Yang et al., 2019) where the same procedure was already successfully used in the past to yield a roughly balanced proportion of positive and negative feedback and promote task engagement despite the inherent variability in RTs across subjects and trials. This cutoff (or RT deadline) was re-set (and identical) at the start of each block, but adapted on a trial-by-trial basis based on the preceding trial (i.e., it corresponded to the average of the current and preceding RT). If the response was correct and fast enough (i.e., faster than current cutoff adjusted on a trial-by-trial basis), then a positive feedback was given; while a negative feedback was given instead if the response was too slow (i.e., correct but slower than the cutoff), or incorrect. With these parameters, the proportion of negative feedback was around 55 – 60% in our previous studies, as well as in the current study (see proportion of negative feedback for each condition and each experiment separately shown in Table 1). Unknown to participants, the RT cutoff was updated on a trial-by-trial basis to deal with unwanted fatigue or habituation effects throughout the experimental session. For the feedback-non contingent conditions, negative and positive feedback were pre-set and shown in random order according to the proportions found in the Feedback-contingent conditions (i.e., 55 – 60%; see Table 1).

**TABLE 1:** Proportion (expressed in percentage) of negative feedback for each condition and each experiment separately.

Condition types	PC	PNC	NC	NNC
Experiment 1	57.38%	56.56%	59.01%	59.26%
Experiment 2	58.10%	56.38%	60.77%	58.02%

After having signed the informed consent, participants started with a short practice session that consisted of two blocks of 16 trials each, without any punishment involved, corresponding to NC and NNC conditions. Afterward, they moved to the experimental session that consisted of 12 blocks, some of which included punishment (PC and PNC conditions) while the other ones did not (NC and NNC conditions). Each condition (i.e., PC, PNC, NC, and NNC) had three successive blocks, and the order of conditions was counterbalanced across participants. Each block included 81 trials. Stimuli were shown in a pseudo-random order and the same number of congruent vs. incongruent trials was used. Self-spaced breaks were allowed in between blocks. Prior to each of them, participants were given written instructions about the involvement of punishment or not, but also about feedback’s contingency, namely whether it was contingent on task performance or random. For all conditions, they were encouraged to be accurate and fast. After the practice session and after each condition (i.e., at the end of the three blocks of each condition), subjective feelings (PANAS and dislike feelings; see here below) were reported by the participants. Stimuli presentation and data recording were controlled using E-Prime (Version 2.0; Psychology Software Tools Inc., Sharpsburg, PA, USA).

#### Subjective ratings

##### PANAS

A Dutch version of the Positive and Negative Affect Schedule (PANAS; Watson et al., 1988; Engelen et al., 2006) was used to measure changes in affect across the four main conditions (PC, PNC, NC, and NNC). The scale consists of 20 words describing different feelings and emotions (10 items for negative affect, 10 items for positive affect). Participants were asked to report their subjective feelings by rating these items on a 5-point scale ranging from 1 – Very slightly or not at all to 5 – Extremely. The order of these 20 items was changed across the 5 measurement points (1 for the baseline after the practice session, and the other 4 corresponded to four main conditions) to avoid the use of a specific response strategy. The PANAS served as main manipulation check regarding the (expected) increase in negative affect when punishment was involved (i.e., in the PC and PNC conditions).

##### Dislike feelings (negative feedback)

Participants were also asked to rate their feelings toward the negative feedback by means of a Visual Analog Scale (VAS) ranging from 0 (not at all) to 100 (a lot) along a dislike continuum. These ratings served as additional manipulation check to corroborate the increase in negative affect when punishment was involved (i.e., PC and PNC conditions).

#### Data analysis

##### Manipulation checks

###### PANAS

The values of negative and positive affect were obtained from the sum of scores on negative and positive items, respectively. The resulting PANAS values were analyzed by means of an ANOVA with Feedback type (punishment vs. neutral), Feedback contingency (contingent vs. non-contingent), and Affect (negative vs. positive) as within-subject factors^2^.

###### Dislike feelings (negative feedback)

The ratings for the negative feedback were analyzed by means of an ANOVA with Feedback type (punishment vs. neutral) and Feedback contingency (contingent vs. non-contingent) as within-subject factors.

##### Behavioral data analysis

Data preprocessing, visualization and analysis were carried out in R (R Core Team, 2018), using the tidyverse (Wickham et al., 2019), ggplot2 (Wickham et al., 2016), lme4 (Bates et al., 2015), lmerTest (Kuznetsova et al., 2017), emmeans (Lenth et al., 2019), reshape2 (Wickham, 2007), and ggiraphExtra (Moon, 2018) packages. For each subject separately, outlier trials (values falling ± 3SDs above/below the mean) were excluded from the data analysis (accuracy). The accuracy data were analyzed using a generalized linear mixed model (GLMM) with binomial distribution and a logit link function. For the RT data analysis, error trials and outliers (values falling ± 3SDs above/below the mean) were excluded. They were analyzed using a linear mixed model (LMM) with which RTs were log-transformed.

The full model was created based on three factors (i.e., Congruency, Feedback type, and Feedback contingency). Accordingly, the three main effects, the three two-way interactions, and the three-way interaction were added as fixed effects. Moreover, in order to investigate whether negative affect could influence conflict processing in combination with aversive motivation or not, we added it as an additional factor in the full model and tested whether it could predict task performance at the RT and accuracy levels. More specifically, we added levels of negative affect (from the PANAS) of each condition (i.e., PC, PNC, NC, and NNC) for each subject into the model. Accordingly, the full model included four factors (i.e., Congruency, Feedback type, Feedback contingency, and Negative affect). For the LMM (RTs) and GLMM (ACC), participant was added as the random effect. For each analysis, we compared the model with negative affect to the model without it, and selected the best one each time (i.e., best-fit model). Moreover, in order to assess effects of interest (i.e., the main and interaction effects) of the selected model (either with or without negative affect), we compared models with and without these fixed effects of interest using likelihood ratio tests. For each comparison, the model included all other fixed effects that would conceivably influence the data, as well as identical random effects structures. We used the mean-centered deviation coding for these factors.

#### Results

##### Manipulation checks

###### PANAS

**FIGURE 2:**
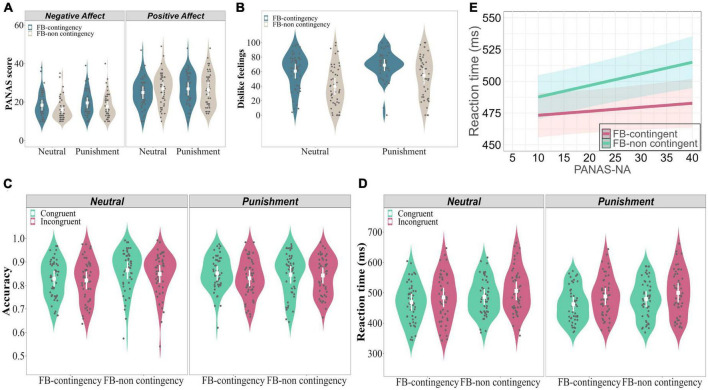
Subjective ratings and behavioral results of Experiment 1. PANAS **(A)** and negative feedback ratings **(B)** both confirmed that negative affect increased with the involvement of punishment, but also when contingency was created between the response and the feedback. Not only negative affect increased with these two manipulations, but also positive affect decreased symmetrically. **(C)** Accuracy results. In the Neutral conditions, Congruency did not significantly interact with Feedback contingency. In the Punishment conditions, accuracy was significantly higher for congruent than incongruent trials in the Feedback-contingent conditions, while it was comparable for them in the Feedback-non contingent conditions. **(D)** RTs results. In the Neutral conditions, the gain in RTs for congruent compared to incongruent trials was comparable for the Feedback-contingent and the Feedback-non contingent conditions. However, when punishment was involved, this difference was larger in the Feedback-contingent than the Feedback-non contingent conditions. **(E)** RTs were slower with higher levels of NA, mostly in the Feedback-non contingent conditions relative to the Feedback-contingent ones. In these graphs, the mean value and standard error (SE) are shown in white, while individual subject data are shown using black dots.

The ANOVA showed a significant three-way interaction between Feedback type, Feedback contingency, and Affect, *F*(1, 49) = 4.574, *p* = 0.037, η^2^ = 0.002. For negative affect (Figure 2A, left panel), main effects of Feedback type and Feedback contingency were significant (*Fs* ≥ 10.922, *ps ≤* 0.002, η^2^s ≥ 0.070). Negative affect increased in Punishment compared to Neutral conditions, and in the Feedback-contingent relative to the Feedback-non contingent conditions. In contrast, for positive affect (Figure 2A, right panel), the two factors (i.e., Feedback type and Feedback contingency) significantly interacted with each other (*F*(1, 49) = 4.552, *p* = 0.038, η^2^ = 0.028). Positive affect was significantly higher in the Feedback-non contingent than the Feedback-contingent conditions without punishment (t(49) = 2.537, *p* = 0.014, Cohen’s d = 0.359), while no such difference was found when punishment was used (t(49) = 0.419, *p* = 0.677, Cohen’s d = 0.059).

###### Dislike feelings (negative feedback)

The ANOVA showed a significant main effect of Feedback type, *F*(1, 49) = 21.171, *p <* 0.001, η^2^ = 0.089, with higher dislike feelings found for the Punishment relative to the Neutral conditions (Figure 2B). The main effect of Feedback contingency was also significant, *F*(1, 49) = 37.236, *p <* 0.001, η^2^ = 0.204, with higher dislike feelings in the Feedback-contingent than the Feedback-non contingent conditions. The interaction between these two factors was not significant, *F*(1, 49) = 1.556, *p* = 0.218, η^2^ = 0.007.

##### Behavioral data

###### Accuracy

The model comparison showed that the model with negative affect was marginally significantly better than the one without it (χ^2^(8) = 15, *p* = 0.059). Accordingly, the model with four factors (Congruency, Feedback type, Feedback contingency, and Negative affect) was selected. Results showed a significant main effect of Congruency, χ^2^(1) = 13.71, *p* = 0.0002, indicating a higher accuracy for Congruent than Incongruent trials. The main effect of Feedback contingency was also significant, χ^2^(1) = 32.806, *p <* 0.0001, indicating a higher accuracy in the Feedback-non contingent relative to the Feedback-contingent conditions. The main effect of Negative affect was also significant, χ^2^(1) = 7.59, *p* = 0.006, indicating increased accuracy overall with higher levels of Negative affect. In addition, the two-way interaction between Feedback type and Feedback contingency was significant, χ^2^(1) = 19.56, *p* < 0.0001. It indicated that in the Neutral conditions, accuracy was significantly higher in the Feedback-non contingent than Feedback-contingent conditions (*z* = 7.027, *SE* = 0.04, *p <* 0.0001); whereas this difference was not significant in the Punishment conditions (*z* = 1.250, *SE* = 0.038, *p* = 0.211). Moreover, the three-way interaction between Congruency, Feedback type, and Feedback contingency was significant, χ^2^(1) = 4.879, *p* = 0.027. To further explore this significant three-way interaction, two GLMMs including two factors (Congruency and Feedback contingency) were computed, for the Punishment and Neutral conditions separately. In the Neutral conditions (Figure 2C, left panel), the main effect of Feedback contingency was significant, χ^2^(1) = 47.224, *p <* 0.0001, with a higher accuracy in the Feedback-non contingent than the Feedback-contingent conditions. However, the two-way interaction between Congruency and Feedback contingency was not significant, χ^2^(1) = 1.365, *p* = 0.243. In contrast, in the Punishment conditions (Figure 2C, right panel), the two-way interaction between Congruency and Feedback contingency was marginally significant, χ^2^(1) = 3.732, *p* = 0.05. In the Feedback-contingent conditions, accuracy was significantly higher for congruent than incongruent trials (*z* = 3.204, *SE* = 0.05, *p* = 0.001); whereas this difference was not significant in the Feedback-non contingent conditions (*z* = 0.456, *SE* = 0.05, *p* = 0.649).

##### RTs

The model comparison showed that the model with negative affect was significantly better than the one without it (χ^2^(8) = 45.259, *p <* 0.0001; see Table 2). Thus, the model including four factors (Congruency, Feedback type, Feedback contingency, and Negative affect; also see Table 2) was selected. Results showed a significant main effect of Congruency, χ^2^(1) = 317.6, *p* < 0.0001, with faster RTs for Congruent than Incongruent trials. The main effect of Feedback type was also significant, χ^2^(1) = 30.34, *p* < 0.0001, with faster RTs in the Punishment than Neutral conditions. The main effect of Feedback contingency was also significant, χ^2^(1) = 306.54, *p* < 0.0001, with faster RTs for Feedback-contingent than Feedback-non contingent conditions. Moreover, the main effect of Negative affect was also significant, χ^2^(1) = 18.52, *p* < 0.0001, with slower responses with higher levels of Negative affect. In addition, the two-way interaction between Feedback contingency and Negative affect was significant, χ^2^(1) = 30.025, *p* < 0.0001, indicating that in the Feedback-non contingent conditions (see green line in Figure 2E), this effect (i.e., slower RTs with higher levels of Negative affect) was larger than in the Feedback-contingent conditions (see red line in Figure 2E). Importantly, the three-way interaction between Congruency, Feedback type, and Feedback contingency was significant, χ^2^(1) = 4.721, *p* = 0.030. To further explore it, two LMMs including two factors (Congruency and Feedback contingency) were computed, for the Punishment and Neutral conditions separately. In the Neutral conditions (Figure 2D, left panel), the two-way interaction between Congruency and Feedback contingency was not significant, χ^2^(1) = 0.728, *p* = 0.394. In contrast, in the Punishment conditions (Figure 2D, right panel), the two-way interaction between Congruency and Feedback contingency was significant, χ^2^(1) = 4.304, *p* = 0.038. It indicated that the gain in RTs for congruent compared to incongruent trials was larger in the Feedback-contingent (*z* = 11.864, *SE* = 0.002, *p <* 0.0001) than the Feedback-non contingent conditions (*z* = 8.938, *SE* = 0.002, *p <* 0.0001). However, Negative affect did not influence this pattern and/or interact with other factors, χ^2^s ≤ 2.195, *ps* ≥ 0.139.

**TABLE 2:** Results for model comparisons and summary of fixed effects for the analysis based on RTs (Experiment 1).

Fixed model	AIC	BIC	χ ^2^	Pr (>χ ^2^)
FBType × FBContingency × Congruency	−76248	−76162	–	–
FBType × FBContingency × Congruency × NA	−76277	−76122	45.259	3.287e−07***
**Selected model = [RT ∼ FBType × FBContingency × Congruency × NA + (1| Subject)]**				
Predictor	Estimate	SE	*t*-value	Pr (>| z|)
FBType	−5.46e−03	9.91e−04	−5.51	3.61e−08***
FBContingency	−1.84e−02	1.05e−03	−17.542	<2e−16***
Congruency	−1.70e−02	9.53e−04	−17.853	<2e−16 ***
NA	9.31e−04	2.16e−04	4.311	1.63e−05 ***
FBType:FBContingency	6.22e−03	1.91e−03	3.252	0.001 **
FBType:Congruency	−4.74e−03	1.91e−03	−2.484	0.013 *
FBContingency:Congruency	−1.90e−03	1.91e−03	−0.995	0.320
FBType:NA	−5.00e−06	1.54e−04	−0.031	0.975
FBContingency:NA	−8.47e−04	1.55e−04	−5.481	4.26e−08 ***
Congruency:NA	2.22e−04	1.50e−04	1.481	0.139
FBType:FBContingency:Congruency	−8.28e−03	3.81e−03	−2.172	0.030 *
FBType:FBContingency:NA	3.64e−04	3.06e−04	1.189	0.234
FBType:Congruency:NA	2.82e−04	3.00e−04	0.939	0.348
FBContingency:Congruency:NA	4.60e−05	3.00e−04	0.153	0.879
FBType:FBContingency:Congruency:NA	−3.30e−04	6.00e−04	−0.549	0.583

***<0.001; **<0.01; *<0.05. FBType, Feedback Type; FBContingency, Feedback Contingency; NA, Negative affect.

### Discussion

Results of Experiment 1 showed that both punishment and feedback contingency substantially increased negative affect at the subjective level, and these two effects were independent from each other. This was observed for both the PANAS as well as dislike feelings toward the negative feedback using a VAS. Accordingly, not only punishment *per se*, but also the contingency created between the actual performance and the feedback increased negative affect. However, this increase in negative affect at the subjective level as a function of punishment and feedback contingency did not lead to a clear gain in conflict processing, suggesting a likely dissociation between them. Although punishment and contingency made participants responded faster on average, they did not solve conflict (i.e., incongruent trials) faster (and/or better) in these conditions compared to the neutral conditions and/or when the feedback was not contingent on task performance.

Although it remains difficult to explain this lack of modulation of conflict processing as a function of aversive motivation, there may be some methodological factors that could have prevented it from occurring. Since “by design” punishment had a high probability (i.e., 55%) and moreover this probability was similar across all four conditions, we could argue that participants perhaps did not clearly perceive the difference between them, in particular between the feedback contingent and feedback-non contingent conditions. As a result, they could not use feedback contingency to guide and sharpen conflict processing, especially when punishment was involved. In this scenario, conflict processing did not benefit clearly from the combination of punishment with feedback contingency because participants could not really “feel” or “sense” it to some extent. Hence, metacognition, which is an important determinant of cognitive control (Lai, 2011; Hommel and Wiers, 2017; Desender et al., 2021b), might potentially account for the lack of modulation of conflict processing by punishment and feedback contingency found in this experiment. Relatedly, awareness of feedback contingency, which was not measured and considered in this experiment, could be an important factor to consider when modulations of conflict processing by means of aversive motivation or negative affect are expected. To test this possibility more explicitly, we ran the same experiment in a new sample of participants. However and crucially, we also measured at the subjective level their awareness of feedback contingency and used this information to analyze the task data and eventually assess whether conflict processing could benefit from punishment when it is contingent on task performance, and the participants were aware of this association.

## Experiment 2

Experiment 2 was identical to Experiment 1, except that we also measured at the subjective level the awareness of the contingency created between the response and the feedback created in some conditions, but not other ones. We aimed to replicate the results of Experiment 1 when awareness of feedback contingency was not considered in the analyses of the task data. However and importantly, we also assessed whether conflict processing could benefit from punishment contingent on task performance when participants were aware of this specific association.

### Methods

#### Participants

The sample size was comparable to that of Experiment 1. Fifty participants were recruited through flyers posted in a WeChat group from China Sichuan Normal University. Three of them were excluded because of low accuracy (i.e., smaller than 60%). As a result, forty-seven participants were included in the final data analyses (3 males, mean age = 19.60 years, SD = 1.30). They all had normal or corrected-to normal vision, and no history of psychiatric or neurological disorders. On average, participants lost 5 RMB during blocks with punishment feedback and were compensated 25 RMB for their participation.

#### Stimuli and task

The stimuli and the task were the same as those used in Experiment 1.

#### Procedure

The procedure was identical to Experiment 1, with the exception that after each condition (i.e., at the end of the three blocks of each condition), participants were also asked to rate their awareness of the contingency created between their response and the feedback.

#### Subjective ratings

##### PANAS, dislike feelings

Similarly to Experiment 1, participants filled in the PANAS (a Chinese version of it; see Qiu et al., 2009), rated the negative feedback by means of a VAS.

##### Awareness of feedback contingency

At the end of each condition (hence 4 times in total), participants’ awareness of the feedback contingency was assessed by asking them to answer a specific question (for a similar procedure, see Desender et al., 2014), namely: “Was the feedback received related to your response?” There were four different response options: “1. I think the feedback was contingent on actual performance”; “2. I don’t know! (but I guess the feedback was contingent on actual performance)”; “3. I don’t know! (but I guess the feedback was not contingent on actual performance)”; or “4. I think the feedback was not contingent on actual performance.”

#### Data analysis

##### Manipulation checks

###### PANAS and dislike feelings

They were analyzed similarly to Experiment 1.

###### Awareness of feedback contingency

Awareness of feedback contingency was measured for each subject after each condition (i.e., PC, PNC, NC, and NNC). Wilcoxon tests were used to compare these values.

##### Behavioral data analysis

Data preprocessing, analysis, and visualization were similarly to Experiment 1. Moreover, we also ran a second analysis where we added awareness of feedback contingency as an additional factor to the model. To this end, we computed a mean awareness score across the four main conditions for each participant. More specifically, for each participant separately, we first reversed the scores for the PNC and NNC conditions before we averaged these values with those obtained for the PC and NC conditions. Accordingly, a lower mean score indicated a larger awareness of feedback contingency.

#### Results

##### Manipulation checks

###### PANAS

**FIGURE 3:**
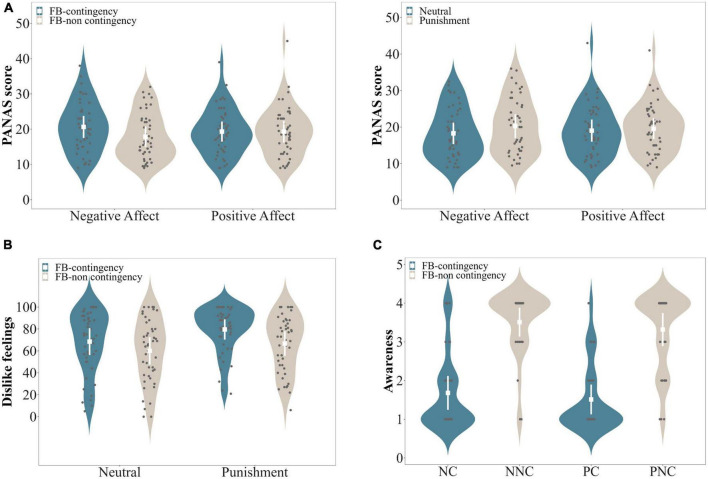
Subjective ratings of Experiment 2. **(A)** PANAS results. Negative affect was significantly higher in the Feedback-contingent than the Feedback-non contingent conditions; and was also significantly higher in the Punishment than the Neutral conditions. Positive affect was comparable between the experimental conditions (Feedback-contingent vs. Feedback-non contingent; Punishment vs. Neutral). **(B)** Dislike ratings of negative feedback were significantly higher in Punishment than Neutral conditions; and were also significantly higher in the Feedback-contingent than the Feedback-non contingent conditions. **(C)** Awareness was larger in the PC than the PNC conditions, and was larger in NC than the NNC conditions (on these graphs, the larger the awareness of feedback contingency, the lower the value on the Y axis). In these graphs, the mean value and standard error (SE) are shown in white, while individual subject data are shown using black dots.

The ANOVA showed a significant two-way interaction between Feedback contingency and Affect, *F*(1, 46) = 11.493, *p* = 0.001, η^2^ = 0.015, as well as a marginally significant two-way interaction between Feedback type and Affect, *F*(1, 46) = 2.958, *p* = 0.092, η^2^ = 0.004. These two interaction effects indicated that Negative affect was significantly higher in the Feedback-contingent than the Feedback-non contingent conditions (t(46) = 4.851, *p* < 0.001, Cohen’s d = 0.708), and was significantly higher in the Punishment than the Neutral conditions (t(46) = 2.614, *p* = 0.012, Cohen’s d = 0.381; Figure 3A). However, no such difference was found for Positive affect (*t*s ≤ 1.023, *ps* ≥ 0.312, Cohen’s ds ≤ 0.149; see Figure 3A). The three-way interaction between Feedback type, Feedback contingency, and Affect was not significant, *F*(1, 46) = 0.630, *p* = 0.431, η^2^ = 0.00007.

###### Dislike feelings (negative feedback)

The ANOVA showed a significant main effect of Feedback type, *F*(1, 46) = 13.254, *p <* 0.001, η^2^ = 0.084, with higher dislike feelings for the Punishment relative to the Neutral conditions (Figure 3B). The main effect of Feedback contingency was also significant, *F*(1, 46) = 19.701, *p <* 0.001, η^2^ = 0.122, with higher dislike feelings in the Feedback-contingent than the Feedback-non contingent conditions (Figure 3B). The interaction between these two factors was not significant, *F*(1, 46) = 1.170, *p* = 0.285, η^2^ = 0.005.

###### Awareness of feedback contingency

Wilcoxon *t*-tests showed that awareness of feedback contingency was significantly higher in the PC (M = 1.51, SD = 0.88) than the PNC condition (M = 3.32, SD = 0.98; *z* = 5.157, *p* < 0.001). Moreover, it was significantly higher in the NC (M = 1.68, SD = 1) than the NNC condition (M = 3.51, SD = 0.86; *z* = 5.478, *p* < 0.001) (see Figure 3C). The mean value was 1.59 (SD = 0.56, Range: 1–3); please note that a lower mean value denoted a higher awareness of feedback contingency.

##### Behavioral data when awareness of feedback contingency is not considered

###### Accuracy

**FIGURE 4:**
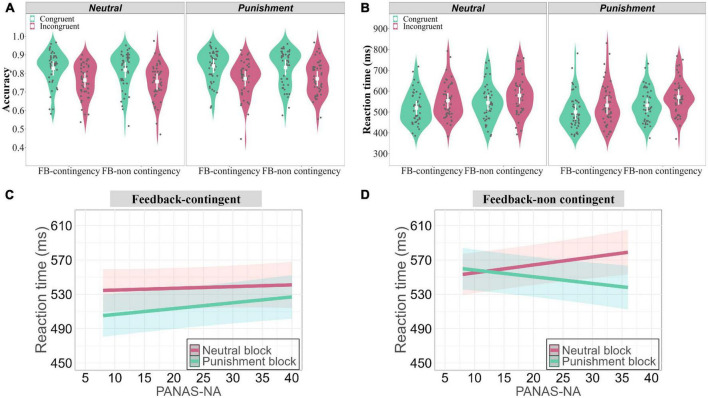
Behavioral results of Experiment 2. **(A)** Accuracy results. **(B)** RTs results. RTs were faster for Congruent than Incongruent trials, faster in the Punishment than Neutral conditions, and faster for the Feedback-contingent than Feedback-non contingent conditions. The two-way interaction between Feedback type and Negative affect was not significant in the Feedback-contingent conditions **(C)**, whereas it was significant in the Feedback-non contingent conditions **(D)**. Colorful areas around the line in panels **(C,D)** represent the 95% confidence interval. In these graphs, the mean value and standard error (SE) are shown in white, while individual subject data are shown using black dots.

The model comparison showed that the model with negative affect did not make a significant contribution compared to the model without it (χ^2^(8) = 10.372, *p* = 0.240). Accordingly, the model including three factors (Congruency, Feedback type, and Feedback contingency) was selected. It revealed a significant main effect of Congruency, χ^2^(1) = 299.94, *p <* 0.0001, indicating a higher accuracy for Congruent than Incongruent trials. The main effect of Feedback type was also significant, χ^2^(1) = 11.251, *p* = 0.0008, indicating a higher accuracy for Punishment than Neutral conditions (see Figure 4A). The three-way interaction between Feedback type, Feedback contingency, and Congruency was not significant, χ^2^(1) = 0.177, *p* = 0.674.

##### RTs

The model comparison showed that the model with negative affect was significantly better than the one without it (χ^2^(8) = 48.335, *p <* 0.0001; also see Table 3). Thus, the model with four factors (Congruency, Feedback type, Feedback contingency, and Negative affect; see Table 3) was selected. Results revealed a significant main effect of Congruency, χ^2^(1) = 661.84, *p* < 0.0001, with faster RTs for Congruent than Incongruent trials. The main effect of Feedback type was also significant, χ^2^(1) = 189.05, *p* < 0.0001, with faster RTs in the Punishment than Neutral conditions. The main effect of Feedback contingency was also significant, χ^2^(1) = 664, *p* < 0.0001, with faster RTs for the Feedback-contingent than the Feedback-non contingent conditions (see Figure 4B). In addition, the two-way interaction between Feedback type and Negative affect was significant, (χ^2^(1) = 8.663, *p* = 0.003), indicating that responses were slower with higher levels of Negative affect in the Neutral but not in the Punishment conditions. Moreover, the three-way interaction between Feedback type, Feedback contingency, and Negative affect was significant (χ^2^(1) = 36.599, *p* < 0.0001). Whereas the two-way interaction between Feedback type and Negative affect in the Feedback-contingent conditions was not significant (χ^2^(1) = 1.251, *p* = 0.263; see Figure 4C), it was in the Feedback-non contingent conditions (χ^2^(1) = 51.715, *p* < 0.0001). In these latter conditions, RTs were faster in the Punishment conditions with increased levels of Negative affect, while they were slower in the Neutral conditions with increased levels of Negative affect (see Figure 4D). However, Negative affect did not influence this pattern and/or interact with the other factors, χ^2^s ≤ 0.154, *ps* ≥ 0.695.

**TABLE 3:** Results for model comparisons and summary of fixed effects for the analysis based on RTs (Experiment 2).

Fixed model	AIC	BIC	χ ^2^	Pr (>χ ^2^)
FBType × FBContingency × Congruency	−65872	−65787	–	–
FBType × FBContingency × Congruency × NA	−65904	−65751	48.335	8.525e−08 ***
**Selected model = [RT ∼ FBType × FBContingency × Congruency × NA + (1| Subject)]**				
Predictor	Estimate	SE	*t*-value	Pr (>| z|)
FBType	−1.48e−02	1.07e−03	−13.765	<2e−16 ***
FBContingency	−2.79e−02	1.10e−03	−25.486	<2e−16 ***
Congruency	−2.71e−02	1.05e−03	−25.838	<2e−16 ***
NA	2.37e−04	1.38e−04	1.716	0.086.
FBType:FBContingency	−1.20e−02	2.10e−03	−5.719	1.08e−08 ***
FBType:Congruency	3.76e−04	2.10e−03	0.179	0.858
FBContingency:Congruency	3.10e−03	2.10e−03	1.478	0.140
FBType:NA	−4.28e−04	1.45e−04	−2.943	0.003 **
FBContingency:NA	4.50e−05	1.41e−04	0.318	0.750
Congruency:NA	2.70e−05	1.37e−04	0.197	0.844
FBType:FBContingency:Congruency	4.67e−03	4.19e−03	1.113	0.266
FBType:FBContingency:NA	1.73e−03	2.85e−04	6.05	1.46e−09 ***
FBType:Congruency:NA	1.08e−04	2.74e−04	0.393	0.695
FBContingency:Congruency:NA	7.30e−05	2.74e−04	0.265	0.791
FBType:FBContingency:Congruency:NA	−1.74e−04	5.48e−04	−0.317	0.751

***<0.001; **<0.01; .<0.1. FBType, Feedback Type; FBContingency, Feedback Contingency; NA, Negative affect.

##### Behavioral data when awareness of feedback contingency is considered

For these analyses, Negative affect was not included in the model because the results presented here above clearly showed that it did not influence conflict processing (i.e., Congruency). Hence, the full model included four factors (Congruency, Feedback type, Feedback contingency, and Awareness).

##### Accuracy

The model comparison based on the fixed effects confirmed that the main effects of Congruency and Feedback type were both significant, χ^2^s ≥ 11.212, *ps* ≤ 0.0008. The main effect of Feedback contingency, as well as the three way-interaction between Feedback type, Feedback contingency, and Awareness were marginally significant, χ^2^s ≥ 3.045, *ps* ≤ 0.081. We did not observe any other significant effect, χ^2^s ≤ 1.352, *ps* ≥ 0.245.

##### RTs

The model (see Table 4) showed that the main effects of Congruency, Feedback type, and Feedback Contingency were all three significant, χ^2^s ≥ 162.17, *ps* < 0.0001. In addition, the main effect of Awareness was significant, χ^2^(1) = 6.099, *p* = 0.014, with faster RTs with increased awareness (i.e., smaller scores). Moreover, the three-way interaction between Feedback type, Feedback contingency, and Awareness was significant, χ^2^(1) = 24.035, *p* < 0.0001; as was the three-way interaction between Feedback contingency, Congruency, and Awareness, χ^2^(1) = 4.587, *p* = 0.032. Importantly, the four-way interaction between Feedback type, Feedback contingency, Congruency and Awareness was also significant, χ^2^(1) = 4.791, *p* = 0.029.

**TABLE 4:** Summary of fixed effects for the analysis based on RTs when the statistical model include awareness of feedback contingency (Experiment 2).

Predictor	Estimate	SE	*t*-value	Pr (>|t|)
FBType	−1.302e−02	1.022e−03	−12.749	<2e−16 ***
FBContingency	−2.646e−02	1.021e−03	−25.903	<2e−16 ***
Congruency	−2.702e−02	1.022e−03	−26.429	<2e−16 ***
Awareness	3.976e−02	1.558e−02	2.552	0.014 *
FBType:FBContingency	−1.310e−02	2.043e−03	−6.413	1.44e−10 ***
FBType:Congruency	2.193e−04	2.044e−03	0.107	0.915
FBContingency:Congruency	3.052e−03	2.044e−03	1.493	0.135
FBType:Awareness	−1.671e−03	1.820e−03	−0.918	0.359
FBContingency:Awareness	1.691e−02	1.820e−03	9.292	<2e−16 ***
Congruency:Awareness	−1.638e−03	1.822e−03	−0.899	0.369
FBType:FBContingency:Congruency	5.056e−03	4.089e−03	1.237	0.216
FBType:FBContingency:Awareness	1.785e−02	3.640e−03	4.903	9.46e−07 ***
FBType:Congruency:Awareness	−2.949e−03	3.643e−03	−0.810	0.418
FBContingency:Congruency:Awareness	−7.802e−03	3.643e−03	−2.142	0.032 *
FBType:FBContingency:Congruency:Awareness	−1.595e−02	7.286e−03	−2.189	0.029 *
**Punishment conditions**				
Predictor	Estimate	SE	*t*-value	Pr (>| t|)
FBType	−3.283e−02	1.409e−03	−23.303	<2e−16 ***
Congruency	−2.715e−02	1.410e−03	−19.256	<2e−16 ***
Awareness	3.895e−02	1.633e−02	2.385	0.021 *
FBType:Congruency	5.386e−03	2.819e−03	1.910	0.056 *
FBType:Awareness	2.580e−02	2.517e−03	10.251	<2e−16 ***
Congruency:Awareness	−3.268e−03	2.519e−03	−1.297	0.194
FBType:Congruency:Awareness	−1.578e−02	5.037e−03	−3.133	0.002 **
**Feedback-contingent conditions [The fixed effect: Congruency − Awareness]**				
Congruency	−2.501e−02	2.005e−03	−12.477	<2e−16 ***
Awareness	5.208e−02	1.728e−02	3.014	0.004 **
Congruency:Awareness	−1.105e−02	3.590e−03	−3.078	0.002 **
**Feedback-non contingent conditions [The fixed effect: Congruency − Awareness]**				
Congruency	−2.970e−02	1.913e−03	−15.527	<2e−16 ***
Awareness	2.589e−02	1.600e−02	1.618	0.112
Congruency:Awareness	4.758e−03	3.409e−03	1.396	0.163
**Neutral conditions**				
Predictor	Estimate	SE	*t*-value	Pr (>| t|)
FBType	−1.984e−02	1.470e−03	−13.496	<2e−16 ***
Congruency	−2.706e−02	1.471e−03	−18.401	<2e−16 ***
Awareness	4.073e−02	1.560e−02	2.612	0.012 *
FBType:Congruency	8.479e−04	2.941e−03	0.288	0.773
FBType:Awareness	7.675e−03	2.611e−03	2.939	0.003 **
Congruency:Awareness	−2.251e−04	2.613e−03	−0.086	0.931
FBType:Congruency:Awareness	1.138e−04	5.225e−03	0.022	0.983

***<0.001; **<0.01; *<0.05. FBType, Feedback Type; FBContingency, Feedback Contingency.

**FIGURE 5:**
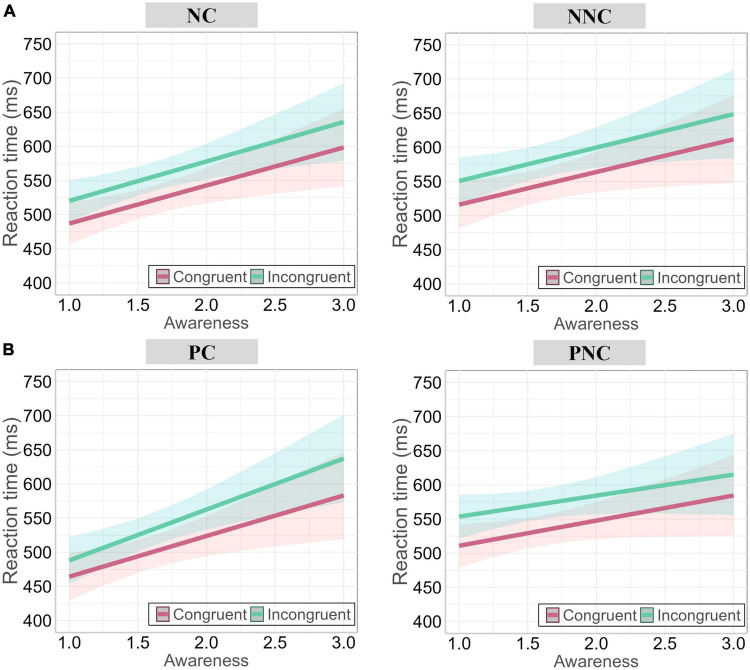
Results of Experiment 2 (RTs) when awareness of feedback contingency is considered and modeled. **(A)** In the Neutral conditions (NC and NNC conditions), awareness of feedback contingency did not influence conflict processing. **(B)** In the Punishment conditions (PC and PNC conditions), awareness of feedback contingency speeded up RTs when the feedback was contingent, selectively. Importantly, in this condition, it did improve conflict processing. Each dot in represents the data of a single trial. Colorful areas around the line in panels **(A,B)** represent the 95% confidence interval.

To further explore it, two LMMs including three factors (Congruency, Feedback contingency, and Awareness) were computed, for the Punishment and Neutral conditions separately. In the Neutral conditions (Figure 5A), the main effects of three factors were all significant; whereas the three-way interaction between them was not significant, χ^2^(1) = 0.0005, *p* = 0.983 (see Table 4: Neutral conditions). In contrast, in the Punishment conditions (Figure 5B and Table 4: Punishment conditions), it was significant, χ^2^(1) = 9.815, *p* = 0.0017. More specifically, in the Feedback-contingent conditions (Figure 5B, left panel), the two-way interaction between Congruency and Awareness was significant, χ^2^(1) = 9.471, *p* = 0.002. It translated faster RTs when Awareness increased and this facilitation was stronger for incongruent (green line, slope = 0.058) than congruent (red line, slope = 0.047) trials (*z* = 3.078, *SE* = 0.004). However, in the Feedback-non contingent conditions (Figure 5B, right panel), the two-way interaction between Congruency and Awareness was not significant (χ^2^(1) = 1.948, *p* = 0.163).

### Discussion

Similarly to Experiment 1, the subjective ratings of Experiment 2 showed that both punishment and feedback contingency increased negative affect, as observed by the PANAS and dislike feelings toward the negative feedback. Moreover, in agreement with Experiment 1, we did not find an improvement of conflict processing as a function of punishment and its contingency on task performance (see summary statistics for each condition and experiment separately in Table 5). However, such a gain was observed when awareness of feedback contingency was considered and directly used as (fixed) factor to analyze the behavioral data. More specifically, when participants were aware of the feedback contingency, task performance improved (as shown by faster RTs), and this gain was greater for incongruent (i.e., conflict) relative to congruent trials. Accordingly, these findings suggest that awareness of feedback contingency is an important factor to consider when effects of aversive motivation on cognitive control are scrutinized.

**TABLE 5:** Summary statistics for each condition and experiment separately. Mean RTs (and standard error of the mean in parenthesis) are shown.

	PC		PNC		NC		NNC	
	C	I	C	I	C	I	C	I
Experiment 1	463.95 (15.34)	488.98 (16.95)	482.89 (16.11)	502.68 (17.56)	472.52 (16.52)	487.72 (17.77)	489.93 (16.10)	508.91 (17.80)
Experiment 2	500.25 (19.65)	530.30 (21.81)	531.96 (18.82)	572.34 (20.80)	520.36 (19.52)	555.01 (21.23)	546.08 (20.93)	580.46 (21.39)

C, congruent trials; I, incongruent trials.

## General discussion

The goal of the current study was to determine if aversive motivation, or rather negative affect, is eventually responsible for the improvement in conflict processing when punishment is involved. To this end, in two experiments, we employed the confound minimized Stroop task to assess conflict processing, while manipulating negative affect and aversive motivation concurrently using a factorial design. More specifically, regarding the former component, slow or incorrect responses during the Stroop task led to monetary loss (punishment condition) or no consequences (neutral condition), and this information was conveyed to the participants by means of an evaluative feedback following each and every response. With regard to the latter one, this feedback was related to actual performance (contingent condition) or not (non-contingent condition). Importantly, the amount of negative vs. positive feedback was balanced between these four conditions. Although the two experiments had a similar design and structure, we also measured the awareness of feedback contingency at the subjective level in Experiment 2 since we figured based on the results of Experiment 1 that this could be an important factor to consider when effects of aversive motivation on cognitive control are explored.

At the subjective level, the results of both experiments converged and confirmed that these two manipulations were successful. Participants reported higher levels of negative affect when punishment was used as well as when the feedback was contingent on task performance, with these two effects being additive. These results are compatible with earlier findings showing that negative affect increases at the subjective level when punishment is used (e.g., Lu et al., 2013; Leotti and Delgado, 2014). They also suggest that feedback contingency is able to increase negative affect, probably because in this situation, it carries important information about self-efficacy (Bandura et al., 1999), and if it is challenged by the frequent encounter of negative feedback (as achieved in the these two experiments), a state of mild fear or apprehension is elicited in turn (Severo et al., 2020; Yee et al., 2022). Interestingly, participants did not report lower levels of positive affect when punishment was used and when the feedback was contingent on task performance, suggesting that these two manipulations had a selective impact on negative affect, while leaving positive affect spared.

However, when we analyzed conflict processing at the behavioral level, we did not find, in none of the two experiments conducted, that it simply improved as a function of negative affect and/or feedback contingency. More specifically, although negative affect influenced performance (mostly RT speed) in relation with feedback contingency alone (Experiment 1) or feedback contingency combined (Experiment 2), it did not improve conflict processing, however. Hence, these results suggest a clear dissociation between negative affect on the one hand and conflict processing on the other hand, and as such, they are not immediately compatible with the affective-signaling hypothesis according to which the former one fuels and sharpens the latter one (Dignath et al., 2020).

Unlike negative affect, aversive motivation (Yee et al., 2022) appears to be the key factor able to explain the context-specific changes of conflict processing depending on punishment, as observed in Experiments 1&2. More specifically, when examining conflict processing at the single trial level, neither congruent nor incongruent trials were influenced by feedback contingency when this feedback was not connected to punishment (i.e., in Neutral conditions). However, in Punishment conditions, RTs were faster in performance-contingent relative to non-contingent conditions (yet irrespective of Congruency), revealing that when participants faced monetary losses that directly depended on their self-efficacy, information processing improved in turn, with this gain being general as opposed to confined to conflict processing (i.e., incongruent trials). This finding accords well with the recent theoretical model put forward by Yee et al. (2022), which assumes that the beneficial influence of punishment on cognitive control depends on whether this incentive is instrumental for behavioral performance or not. In Experiments 1&2, feedback contingency was probably an important cue or information conveyed to the participants, who could harness it to adjust or tweak their cognitive control level and this way try to reduce or avoid the encounter of punishment (Di Rosa et al., 2015; Ryan and Deci, 2017), even though by design, it was not possible to improve substantially in this condition compared to the non-contingent feedback one. In this latter condition (PNC), punishment likely operated as an affective event (perhaps increasing annoyance or frustration; see Inzlicht et al., 2015) rather than a strong motivational drive or incentive.

Moreover, it is noteworthy that feedback contingency is beneficial to performance and cognitive control insofar as the task demands are not too high and/or not exceeding current processing capacities at the cognitive level (DePasque and Tricomi, 2015). In agreement with this important assumption, previous studies have shown that feedback contingent on task performance could ease conflict processing (Krebs et al., 2010; Drueke et al., 2012; Yang and Pourtois, 2018) and it contributed to suppress irrelevant information processing (Ferdinand and Czernochowski, 2018). Interestingly, in Experiment 1, we found that when punishment was used and the feedback was contingent on task performance, congruent trials benefited the most (in RT speed) from this combination of factors. Presumably, no benefit was found for incongruent trials (i.e., conflict processing *per se*) in this condition because participants could hardly avoid punishment and high levels of cognitive effort were required to resolve them (Westbrook and Braver, 2015; Inzlicht et al., 2018; Kool and Botvinick, 2018; van der Wel and van Steenbergen, 2018; da Silva Castanheira et al., 2021). In comparison, participants probably expected (more) reward (i.e., positive feedback) or enhanced punishment avoidance for congruent trials (Sandra and Otto, 2018; Sayalı and Badre, 2019), which might explain why these trials eventually benefited the most from the joint activation of punishment (its prospect and/or encounter) and feedback contingency. In this framework, participants not only evaluated how much (monetary) losses they would incur upon slow or incorrect response, but they also likely considered the cost or effort needed to avoid them (Kool and Botvinick, 2013; Silvestrini and Gendolla, 2019). As our new results for Experiment 1 show, this computation led to faster processing of congruent trials (perhaps because reward outweighed cost or effort), while it did not alter incongruent trials (perhaps because the perceived cost or effort outweigh reward).

However, caution is required for this interpretation because we did not observe the same change (for congruent trials) in Experiment 2 when we analyzed the data irrespective of awareness of feedback contingency, and hence similarly to Experiment 1. Tentatively, we suggest that the same cost-benefit analysis was not carried out in Experiment 2 by the participants because they were asked, after each and every condition, about their awareness of feedback contingency, and this additional monitoring component might have yielded different cognitive control levels in that experiment compared to Experiment 1 where they were never asked about this feature. In this context, we could imagine that participants of Experiment 2 employed a different cognitive control “style” or strategy (characterized by enhanced metacontrol; Hommel and Wiers, 2017; see Hommel and Colzato, 2017) compared to those of Experiment 1, and the former one might prevent the use of a rapid, reactive and dynamic change of cognitive control levels depending on Congruency, Punishment and Feedback contingency.

Although speculative, this interpretation is reinforced by the fact that in Experiment 2, we found, as expected, that conflict processing did improve with punishment when it was contingent on task performance, yet when participants were aware of this (important) link. This result therefore confirms that metacontrol is an important determinant of cognitive control and it can influence conflict processing (Miller and Geraci, 2011; Hefer and Dreisbach, 2017; Desender et al., 2021b). In agreement with this notion, previous studies already showed that the metacognitive experience of conflict could influence conflict adaptation (Desender et al., 2014; Questienne et al., 2018). Moreover, it has been shown that the metacognitive evaluation of task difficulty based upon internal bodily signals could determine how much effort is eventually allocated to the task at hand (Desender et al., 2021a). In Experiment 2, we could assume that the evaluative feedback, especially when it was contingent on task performance and punishment-related, acted as a potent external signal (Jocham and Ullsperger, 2009) that some participants could directly harness to improve cognitive control, perhaps by increasing effort expenditure (Carpenter and Huet-Vaughn, 2019). Hence, the results of Experiment 2 suggest that aversive motivation, along with metacognition, can influence and boost cognitive control.

More generally, they also align with some recent studies showing that emotions, when task relevant and leading to a conscious appraisal, can influence cognitive control (Calbi et al., 2022; Mancini et al., 2022; Mirabella et al., 2022). These studies indirectly suggest that the conscious appraisal of emotions (Smith et al., 2018) could be an important factor to consider when assessing their modulatory effects on cognitive control. Although they focused on another component of cognitive control and explored other emotions than the one used in the current study, they suggest some boundary conditions for the modulation of cognitive control by (negative) emotion. As our new results for Experiment 2 show, the conscious appraisal of emotions, and the awareness of the contingency created between feedback and performance more specifically in the present case, appears to account for some of the variability observed in conflict processing across conditions and subjects.

To conclude, across two experiments, we failed to evidence a clear link between increases in negative affect (at the subjective level) and improvements in cognitive control (e.g., conflict processing). Instead, this ability improved the most when punishment was used and the feedback was contingent on task performance, for participants who were aware of this association. Hence, they suggest that aversive motivation (Yee et al., 2022) and metacontrol (Hommel and Wiers, 2017) could interact with each other and eventually influence cognitive control. As such, these new results might help to revise and improve current theoretical models of cognitive control where synergistic effects of metacognition and aversive motivation are usually not considered to account for the context-dependent modulations of this remarkable ability (Shenhav et al., 2013; Dreisbach and Fischer, 2015; Inzlicht et al., 2015; Dignath et al., 2020).

## Data availability statement

The datasets presented in this study can be found in online repositories. The names of the repository/repositories and accession number(s) can be found below: https://osf.io/k6r7t/.

## Ethics statement

The studies involving humans were approved by the Faculty of Psychology and Educational Sciences at Ghent University; Institute of Brain and Psychological Sciences, Sichuan Normal University. The studies were conducted in accordance with the local legislation and institutional requirements. The participants provided their written informed consent to participate in this study.

## Author contributions

QY: conceptualization, data curation, formal analysis, funding acquisition, and writing. SS: data curation and figure plotting. GP: conceptualization and writing—reviewing and editing. All authors contributed to the article and approved the submitted version.

## Footnotes

1 Of note, three different outcomes could lead to negative feedback: (i) slow (correct) response (i.e., response made above the RT cutoff); (ii) response errors (incorrect responses); (iii) omissions (no responses). While the vast majority of negative feedback resulted from the first outcome, we also analyzed the data for each category separately (see Supplementary material).

2 The ratings (PANAS and Dislike feelings) corresponding to the practice session were not included in these analyses because few trials were used. However, we also analyzed them to assess possible changes relative to the experimental session (see Supplementary material).
