# Supplementary material for: Parsing the contributions of negative affect vs. aversive motivation to cognitive control: an experimental investigation
Source: Front Behav Neurosci. 2023 Sep 18;17:1209824. doi: 10.3389/fnbeh.2023.1209824 (PMC10543231; doi:10.3389/fnbeh.2023.1209824)
Supplement: Supplementary file 1 [file Data_Sheet_1.docx]

**Supplementary materials**

We performed additional analyses (1) to ascertain that the punishment feedback was mostly encountered by the participants after correct (but slow) responding and not simply error making; (2) to clarify the direction of the changes in negative and positive affect at the subjective level between practice (where the negative feedback, if encountered, was not punishment related) and test (where it was in some blocks).

1. As a matter of fact, three different outcomes could lead to negative feedback: (i) slow (correct) responses, i.e., response made above the RT cutoff; (ii) response errors (incorrect responses); or (iii) omissions (no responses). Please note that, as can be seen from the revised Table 1 here below, the majority of negative feedback actually concerned the first category and hence participants likely associated it with slow (but correct) responding rather than error making. However, for each category, we analyzed the data (i.e., proportion of negative feedback) by means of a repeated-measures ANOVA with Congruency (congruent vs. incongruent), Feedback type (punishment vs. neutral), and Feedback contingency (contingent vs. non-contingent) as within-subject factors. The results of these new analyses are presented here below, for the two experiments separately.

**Experiment 1:**

1. Regarding slow responses, the results showed that the main effect of Feedback contingency was significant (*F*(1, 49) *=* 181.276 *p <* .001, $\eta^{2}$ = 0.551), with a greater proportion of negative feedback in the Feedback-non contingent than the Feedback-contingent conditions. The two-way interaction between Feedback type and Feedback contingency was also significant (*F*(1, 49) *=* 4.897, *p =* .032, $\eta^{2}$ = 0.007). Moreover, the two-way interaction between Feedback contingency and Congruency was significant (*F*(1, 49) *=* 5.012, *p =* .03, $\eta^{2}$ = 0.003), indicating that in the Feedback-contingent conditions, this proportion was numerally higher for Incongruent (71.4%) than Congruent (70.3%) trials, while it was numerally lower for Incongruent (84.1%) than Congruent (85%) trials in the Feedback-non contingent conditions. However, these two effects did not reach significance.
2. For response errors, the results showed that the main effect of Feedback contingency was significant (*F*(1, 49) *=* 165.615, *p <* .001, $\eta^{2}$ = 0.533), with a greater proportion of negative feedback in the Feedback-contingent than the Feedback-non contingent conditions. The two-way interaction between Feedback type and Feedback contingency was also significant (*F*(1, 49) *=* 4.822, *p =* .033, $\eta^{2}$ = 0.007), indicating that in the Feedback-non contingent conditions, this proportion was slightly higher for Punishment than Neutral conditions (*t*(49) = 1.694, *p =* .097), while it did not differ between the two conditions in the Feedback-contingent conditions (*t*(49) = 1.667, *p =* .102).
3. For omissions, the results showed that the main effect of Feedback contingency was significant (*F*(1, 49) *=* 4.231, *p =* .045, $\eta^{2}$ = 0.023), with a greater proportion of negative feedback in the Feedback-contingent than Feedback-non contingent conditions. The two-way interaction between Feedback contingency and Congruency was also significant (*F*(1, 49) *=* 4.750, *p =* .034, $\eta^{2}$ = 0.007), indicating that this proportion was greater in the Feedback-contingent than Feedback-non contingent conditions for congruent trials (*t*(49) = 2.965, *p =* .005), while it did not differ between the two conditions for incongruent trials (*t*(49) = 0.791, *p =* .433).

**Experiment 2:**

1. For slow responses, the results showed that the main effect of Feedback contingency was significant (*F*(1, 46) *=* 87.590, *p <* .001, $\eta^{2}$ = 0.243), with a greater proportion of negative feedback in the Feedback-non contingent than the Feedback-contingent conditions. The main effect of Congruency was also significant (*F*(1, 46) *=* 41.048, *p <* .001, $\eta^{2}$ = 0.110), with a greater proportion of negative feedback for congruent than incongruent trials. No other effect was significant (*F*s ≤ 2.683, *p*s ≥ .108, $\eta^{2}s$ ≤ 0.007).
2. For responses errors, the results showed that the main effect of Feedback contingency was significant (*F*(1, 46) *=* 92.748, *p <* .001, $\eta^{2}$ = 0.278), with a greater proportion of negative feedback in the Feedback-contingent than the Feedback-non contingent conditions. The main effect of Congruency was also significant (*F*(1, 46) *=* 37.545, *p <* .001, $\eta^{2}$ = 0.097), with a greater proportion of negative feedback for incongruent than congruent trials. No other effect was significant (*F*s ≤ 1.612, *p*s ≥ .211, $\eta^{2}s$ ≤ 0.004).
3. For omissions, the results showed that the main effect of congruency was marginally significant (*F*(1, 46) *=* 3.983, *p =* .052, $\eta^{2}$ = 0.013), with a greater proportion of negative feedback for incongruent than congruent trials.

**SM-Table 1.** The percentage of negative feedback is shown, separately for each outcome leading to it (i.e., slow responses, response errors or omissions).

|  | Slow responses | Response errors | Omissions |
| --- | --- | --- | --- |
| Experiment 1 | 77.7% | 21.6% | 0.7% |
| Experiment 2 | 75.6% | 21.6% | 2.8% |

Taken together, these results and supplementary analyses can be interpreted as follows. First, negative feedback was mostly given after slow (but correct) responses, and not response errors or omission. Second, the proportion of negative feedback was greater in the feedback-non contingent than feedback-contingent conditions when it was driven by (correct) slow responses. Third, the proportion of negative feedback was greater in feedback-contingent than feedback-non contingent conditions, and was greater in incongruent than congruent trials when it was related either to response errors or omissions. All in all, these results therefore suggest that negative feedback was likely perceived and processed differently for slow responses vs. errors. Moreover, we believe it is important to remember that for all RTs data analyses presented in the manuscript, we excluded error trials, which implies that the influence of negative feedback (i.e., punishment and hence aversive motivation) on task performance found for this variable in these two experiments mainly concerned correct (but slow) decisions.

**2.** We also assessed possible changes of NA, PA and dislike feelings towards the negative feedback from the practice session to the test session by conducting planned comparisons (t-tests) for each condition and experiment separately.

**Experiments 1:**

Dislike feelings reported in the Practice session were significantly lower compared to the PC and NC conditions (ts ≥ 2.136, *ps* ≤ .038); whereas they were significantly higher compared to the NNC conditions (t = 2.954, *p* = .005) and comparable to the PNC conditions (t = 0.349, *p* = .729). Levels of NA in the Practice session were significantly lower compared to the PC conditions (t = -2.418, *p* = .019); whereas they were marginally significantly higher compared to the NNC conditions (t = 1.82, *p* = .075), and did not differ from the PNC and NC conditions (ts ≤ 0.975, *ps* ≥ .335). In comparison, levels of PA in the Practice session were significantly higher than any of the four conditions (ts ≥ 5.244, *ps* < .001).

**Experiment 2:**

Dislike feelings and levels of NA in the Practice session were significantly lower, albeit marginally only, compared to the PC conditions (ts ≥ 1.996, *ps* ≤ .052); whereas they were significantly higher compared to the NNC conditions (ts ≥ 3.021, *ps* ≤ .004). Dislike feelings and levels of NA in the Practice session did not significantly differ from the PNC, nor the NC conditions (ts ≤ 1.576, *ps* ≥ .122). In comparison, levels of PA in the Practice session were significantly higher than any of the four conditions (ts ≥ 4.833, *ps* < .001).

SM-Table 2 shown here below presents these values (i.e., mean and standard error of mean) for each condition, scale and experiment separately.

**SM-Table 2.** Levels of NA and PA as well as dislike feelings toward the negative feedback, for each condition and each experiment separately. The mean score (and standard error of mean in parenthesis) is shown.

|  | **Experiment 1** | | | **Experiment 2** | | |
| --- | --- | --- | --- | --- | --- | --- |
|  | PANAS-NA | PANAS-PA | Dislike ratings | PANAS-NA | PANAS-PA | Dislike ratings |
| Baseline | 17.50(0.80) | 32.02(0.76) | 52.92(3.40) | 19.92(0.96) | 24.03(0.90) | 72.77(2.89) |
| PC | 19.56(0.93) | 26.84(1.14) | 68.96(2.77) | 21.77(1.27) | 20.02(1.01) | 79.57(3.08) |
| PNC | 17.56(0.89) | 26.48(1.23) | 54.72(4.05) | 18.77(1.07) | 19.13(1.10) | 66.70(3.75) |
| NC | 18.38(0.92) | 24.92(1.09) | 60.70(3.41) | 19.53(1.05) | 18.62(1.04) | 68.49(4.07) |
| NNC | 16.08(0.80) | 26.96(1.19) | 39.86(3.90) | 17.02(1.08) | 19.43(1.11) | 60.09(4.18) |

Moreover, for each experiment separately, we also performed some exploratory correlational analyses on these subjective ratings. More specifically, we first tested whether NA was correlated with PA for the PANAS. Next, we also tested whether levels of NA and/or PA from the PANAS were correlated with the dislike feelings toward the negative feedback. In Experiment 1, the results did not show any significant correlations (all rs ≤ 0.160, all *ps* ≥ .281). In Experiment 2, results showed that NA was positively correlated with dislike ratings (r = 0.453, *p* = .001); whereas the other correlations were not significant (all rs ≤ 0.195, all *ps* ≥ .188).
